# Ibrutinib in mantle cell lymphoma: a real-world retrospective multi-center analysis of 77 patients treated in the Czech Republic

**DOI:** 10.1007/s00277-022-05023-2

**Published:** 2022-11-11

**Authors:** Ales Obr, Katerina Benesova, Andrea Janikova, Heidi Mocikova, David Belada, Andrea Hruskova, Petra Vockova, David Salek, Alice Sykorova, Tomas Furst, Diana Malarikova, Tomas Papajik, Marek Trneny, Pavel Klener

**Affiliations:** 1 Department of Haemato-Oncology, Faculty of Medicine and Dentistry, Palacky University and University Hospital, Olomouc, Czech Republic; 2 First Department of Internal Medicine–Department of Haematology, University General Hospital and First Faculty of Medicine, Charles University, U Nemocnice 499/2, Prague, 12808 Czech Republic; 3 Department of Haematology and Oncology, University Hospital, Brno, Czech Republic; 4 Department of Internal Medicine and Haematology, Faculty Hospital Kralovske Vinohrady and Third Faculty of Medicine, Charles University, Prague, Czech Republic; 5 4th Department of Internal Medicine–Haematology, University Hospital and Faculty of Medicine, Hradec Kralove, Czech Republic; 6 Department of Mathematical Analysis and Applications of Mathematics, Faculty of Science, Palacky University, Olomouc, Czech Republic; 7 Institute of Pathological Physiology, First Faculty of Medicine, Charles University, Prague, Czech Republic

**Keywords:** Mantle cell lymphoma, Bruton tyrosine kinase, Ibrutinib, Bone marrow, Chemoresistance

## Abstract

Ibrutinib revolutionized therapy for relapsed/refractory (R/R) mantle cell lymphoma (MCL). Real-world data on the outcome of unselected patients are still limited. We analyzed 77 R/R MCL patients receiving ibrutinib with at least one prior systemic anti-lymphoma therapy. After a median follow-up of 14.0 months, 56 patients relapsed/progressed, and 45 died. The overall response rate was 66%, with 31% of complete metabolic remissions on PET/CT. The median progression-free and overall survival (OS) rates were 10.3 and 23.1 months, respectively. The median OS from ibrutinib failure was 3.7 months. High proliferation rate by Ki67 (≥ 30%) and two or more previous therapy lines both negatively correlated with outcome (HR = 2.2, *p* = 0.04, and HR = 2.06, *p* = 0.08, respectively). Female gender borderline correlated with better outcome (HR = 0.53, *p* = 0.08). In multivariate analysis, Ki67 and response to ibrutinib both correlated with OS (*p* < 0.05). Importantly, ibrutinib appeared to better control nodal and extranodal lymphoma than bone marrow (BM) involvement. From 20 patients with detectable BM infiltration (before ibrutinib initiation) achieving complete (*n* = 13) or partial (*n* = 7) metabolic remission, none achieved remission in BM. We confirmed good efficacy of ibrutinib in unselected heavily pre-treated MCL patients. Our findings support the use of a combination of ibrutinib and rituximab in patients with BM involvement.

## Introduction

Mantle cell lymphoma (MCL) is a predominantly aggressive B cell non-Hodgkin lymphoma subtype with an incidence of 1–2 cases/100,000 inhabitants per year [1]. It typically affects elderly patients with a median age at diagnosis of about 65 years and a male to female ratio between 2:1 and 3:1. Most cases demonstrate an aggressive behavior with a historically short median overall survival (OS) of up to 7 years [1–5], but 10–20% of MCL cases comprise indolent variants with superior outcomes [6].

Several prognostic and predictive markers have been described in the literature. The MCL International Prognostic Index (MIPI) and its simplified version (sMIPI) are used as standard prognostic scores to stratify patients into low-, intermediate-, and high-risk groups [7, 8]. The prognostic value of the MIPI was further improved by adding the Ki67 index to the sMIPI [9]. Recently, cytogenetic and molecular aberrations emerged as powerful predictors of the disease behavior, including complex karyotype changes, mutations, and deletions of *TP53*, deletions of *CDKN2A*, or mutations of *KMT2D* [10–15].

Majority of MCL is still considered incurable and it is often characterized by multiple relapses. Treatment strategies have been evolving over years, and the outcomes have significantly improved with implementation of new agents (high-dose cytarabine, bendamustine, rituximab induction as well as maintenance) and optimization of treatment protocols [1, 16–20].

In the last two decades, several innovative agents were introduced into clinical practice that broadened the treatment options in relapsed/refractory (R/R) patients, including bendamustine-based regimens, bortezomib, temsirolimus, lenalidomide, acalabrutinib, and ibrutinib. Ibrutinib is an orally administered Bruton tyrosine kinase inhibitor which blocks B cell proliferation and survival. Due to the high response rate, ibrutinib offers an ideal treatment option for heavily pre-treated MCL patients [21]. Although its efficacy in R/R MCL patients in clinical trials is clearly documented [22–24], real-world data on unselected patients are still insufficient. Patiño-Escobar et al. offered insight into the performance of ibrutinib in unselected patients [25]. However, the studied population was relatively small, and heterogeneous treatment regimens were applied. A recent study by McCulloch et al. involving a large real-world population of MCL patients from the UK confirmed the high efficacy and good safety profile of ibrutinib exclusively in patients in the first relapse [26]. Several other studies have provided evidence on ibrutinib efficacy in pre-treated unselected populations, thus confirming the long-term benefit of ibrutinib in the real world [27–29].

In the present study, a consecutive cohort of MCL patients treated with single-agent ibrutinib in the Czech Republic was retrospectively analyzed to investigate the impact of selected biologic, laboratory, and clinical parameters on their outcome.

## Materials and methods

### Patient selection

Ibrutinib was first approved by the Czech State Institute for Drug Control for R/R MCL patients in 2014. The administration was allowed after approval of reimbursement from a health insurance company for patients not indicated for stem cell transplantation.

This study was a part of the Observational Epidemiological and Clinical Study (NiHiL), ClinicalTrials.gov identifier NCT03199066. All the patients were treated according to the Declaration of Helsinki and provided written informed consent to their anonymous data processing before data entry. The study protocol was approved by the institutional ethics committee of the General University Hospital in Prague (retrospective analysis of the Czech Lymphoma Study Group [CLSG] registry).

In June 2021, all university centers collaborating within the CLSG were asked to identify potentially eligible patients with MCL. Diagnoses of MCL were reviewed by local experienced hematopathologists according to the 2016 revision of the World Health Organization classification of lymphoid neoplasms [30]. Cytomorphological variants were not routinely specified due to a high proportion of cases whose diagnoses were based on bone marrow (BM) trephine biopsy. The inclusion criteria were as follows: age at diagnosis ≥ 18 years, Eastern Cooperative Oncology Group performance status 0–2 [31], at least one prior systemic anti-lymphoma therapy (immunochemotherapy), administration of ibrutinib for a minimum of one day, and commencement by July 2020 at the latest. The database was finally updated and locked in July 2021. A total of 77 patients met the inclusion criteria for analysis; of those, 11 were treated in the RAY and SYMPATICO trials and were enrolled after the study unblinding (ClinicalTrials.gov identifiers: NCT01646021 and NCT03112174).

### Patient evaluation and response assessment

A trephine biopsy and BM aspiration were performed at the time of ibrutinib treatment initiation. BM was examined with immunohistochemistry and/or fluorescence-activated cell sorting (FACS), and the results were revised by a local experienced specialist. The cut-off for FACS confirmation of BM involvement was defined as the presence of at least 5% of clonal cells. Treatment response to ibrutinib was assessed by positron emission tomography (PET/CT) using the 2014 Lugano classification criteria [32]. In patients from clinical trials with ibrutinib therapy initiation before 2014, treatment response was revaluated retrospectively. Treatment failure (relapse or progression) within 12, 24, or 36 months from diagnosis was designated as progression of diseases (POD) POD12, POD24, and POD36, respectively. Representative data for assessment of ibrutinib toxicity were not available due to the retrospective character of the study.

### Statistical analysis

Relations among variables were examined using the Pearson’s non-parametric correlation analysis. Overall survival was defined as the time from ibrutinib treatment initiation to the date of the last follow-up (censored) or the date of death (event) from any cause. Progression-free survival (PFS) was defined as the time from ibrutinib start to relapse, progression, or death from any cause. The effect of predictors on OS and PFS was assessed by means of the semi-parametric Cox regression model. Both univariate and multivariate models were built.

## Results

A total of 77 patients from five Czech university centers met the inclusion criteria for analysis. The patients were diagnosed between November 1997 and December 2019. After a median follow-up of 14.0 months (range: 1.3–92.0 months) from ibrutinib beginning, 26 (34%) patients were alive. The median age at diagnosis was 68 years (range: 40–81 years); the median age at the beginning of ibrutinib treatment was 70 years (range: 42–82 years). Sixty (78%) patients were males.

All but two patients were treated with rituximab-containing regimens in the first line. The median number of previous lines of therapy before ibrutinib treatment was two (range: 1–8). Response to ibrutinib was evaluated at a median of 3 months from therapy initiation (range: 1.3–28.2 months). Overall response, that is complete (CMR) or partial (PMR) metabolic remission, was documented in 47 (66%) patients, with 22 (31%) patients achieving CMR.

Forty-eight out of 73 (66%) patients with available data had BM involvement at the time of diagnosis, and 27 out of 48 (56%) patients had BM involvement at the time of ibrutinib treatment initiation. Twenty patients with detectable BM involvement before ibrutinib initiation who achieved CMR (*n* = 13) or PMR (*n* = 7) according to PET/CT had also BM reassessment (on ibrutinib), some of them repeatedly. Of note, not a single patient achieved remission of MCL in BM; six (30%) patients had reduced MCL load in BM, seven (35%) had stable BM involvement, and another seven (35%) had significantly increased MCL load in BM (while achieving CMR or PMR according to PET/CT). Two patients with no detectable BM involvement before ibrutinib experienced isolated progression in BM (while achieving CMR according to PET/CT). More detailed baseline characteristics of the examined cohort are shown in Table 1.

**Table 1:** Baseline characteristics of the examined cohort

Characteristics	*N* = 77 (%)
Sex, no. male (%)	60 (78)
Median age, years	68 (range 40–81)
Ki67	
≥ 30%	27 (68)
< 30%	13 (33)
N/A	37
Stage (Ann Arbor) before ibrutinib	
I + II	15 (20)
III + IV	60 (80)
N/A	2
Bone marrow involvement before ibrutinib initiation	
Yes	27 (56)
No	21 (44)
N/A	29
MIPI before ibrutinib	
Low risk	18 (25)
Intermediate risk	22 (31)
High risk	32 (44)
N/A	5
First line treatment	
CHOP/CHOP-like	42 (54)
High-dose cytarabine regimen	25 (33)
Non-anthracycline regimen	10 (13)
ASCT	19 (25)
Rituximab maintenance	41 (53)
Treatment response after first line (CT)	
CR/CRu	42 (56)
PR	18 (24)
SD + PD	15 (20)
N/A	2
No. of treatment lines before ibrutinib	
1	22 (29)
2	23 (30)
3	19 (25)
4	6 (8)
5	3 (4)
6 and more	4 (5)
Best response on ibrutinib (PET/CT)	
CMR	22 (31)
PMR	25 (35)
SD + PD	25 (35)
N/A	5

MIPI, Mantle Cell Lymphoma International Prognostic Index; ASCT, autologous stem cell transplant; CR, complete remission; PR, partial remission; SD, stable disease; PD, progressive disease; CMR, complete metabolic remission; PMR, partial metabolic remission

**Fig. 1:**
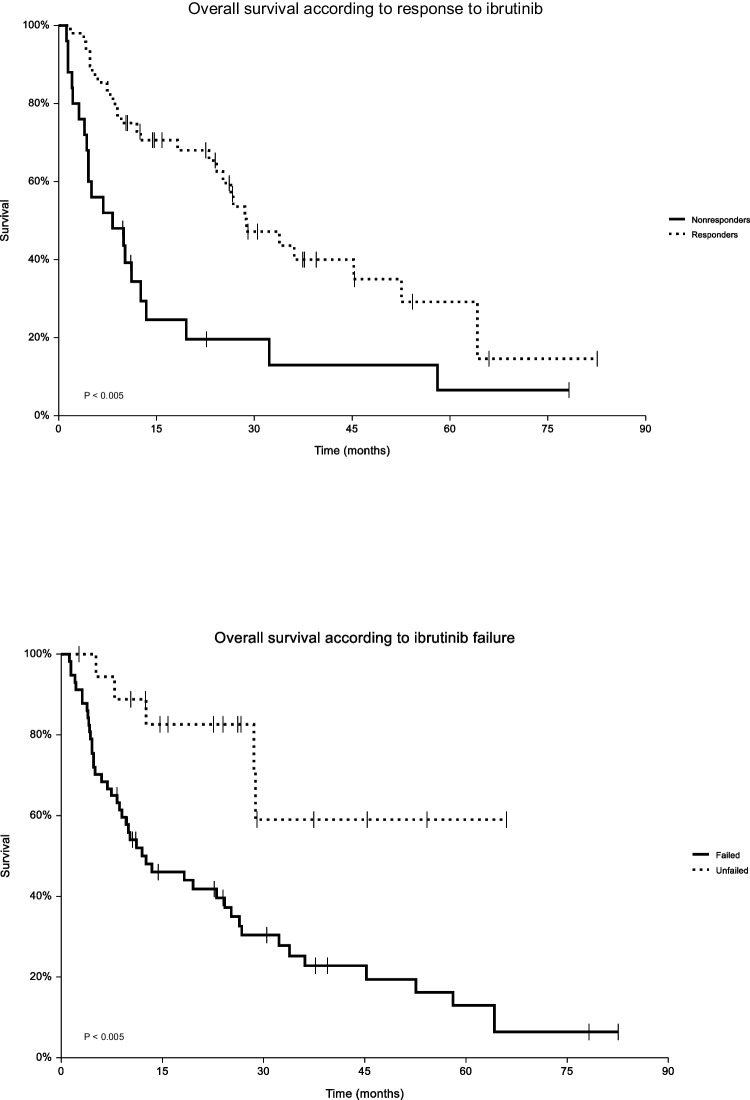
Overall survival of responders vs. nonresponders to ibrutinib and of ibrutinib failed vs. unfailed patients

The median PFS was 10.3 months (range: 1.2–66.0 months), and median OS was 23.1 months (range: 1.2–82.5 months). The median OS of the responders (PMR + CMR) and nonresponders (stable/progressive disease) reached 28.8 months (95% CI 24.2–45.2) and 8.3 months (95% CI 4.3–11.2), respectively (*p* < 0.005). The median OS of the patients who did not relapse on ibrutinib was not reached. The median OS of the patients who relapsed or progressed on ibrutinib reached 12.6 months (95% CI 8.7–24.2) (*p* < 0.005, Fig. 1).

**Fig. 2:**
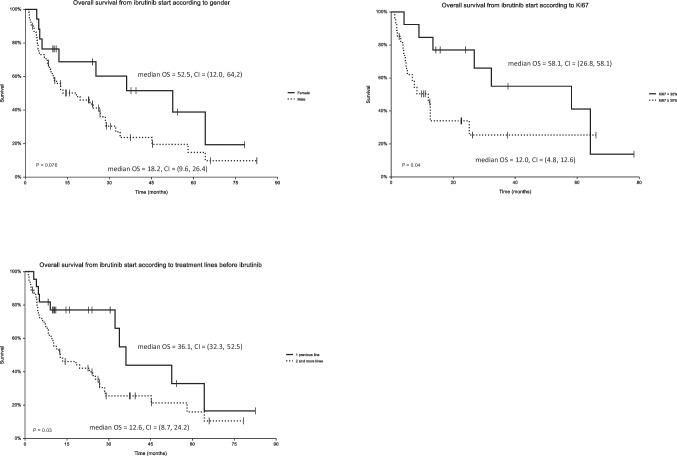
Analyses of overall survival according to gender, Ki67, and treatment lines before ibrutinib

Results of univariate and multivariate analyses are displayed in Table 2 and Fig. 2. Even though all three POD prognostic indexes (i.e., POD12, POD24, and POD36) negatively correlated with overall survival since diagnosis (HR = 2.99, 2.87, and 2.08, respectively, *p* < 0.05), none of them correlated with survival after start of ibrutinib. High proliferation rate by Ki67 (≥ 30%) was associated with shorter OS (HR = 2.20, *p* = 0.04). Patients with one previous line of therapy (before ibrutinib) had significantly longer OS (36.1 months, 95% CI 32.3–52.5) compared to those with two or more previous treatment lines (12.6 months, 95% CI 8.7–24.2, *p* = 0.03). Interestingly, female gender borderline correlated with longer OS (HR = 0.53, *p* = 0.08.)

**Table 2:** Results of univariate and multivariate analyses

Predictor	Hazard ratio (HR)	95% confidence interval (CI)	*p* value
Univariate Cox regression analysis for overall survival calculated from ibrutinib initiation			
Female sex (versus male)	0.53	0.29, 0.98	0.076
Ki67 ≥ 30% (versus < 30%)	2.2	1.00, 4.82	*0.04*
Ann Arbor clinical stage before ibrutinib			
I	Reference		
II	1.05	0.28, 3.89	0.9
III	0.72	0.18, 2.93	0.6
IV	1.14	0.43, 3.04	0.8
MIPI			
Low risk	Reference	—	
Intermediate risk	1.07	0.37, 3.08	0.9
High risk	0.95	0.38, 2.34	0.9
First line regimen			
CHOP/CHOP-like	Reference		
High-dose cytarabine regimen	0.70	0.38, 1.27	0.2
Non-anthracycline regimen	0.79	0.37, 1.71	0.6
ASCT, yes (versus no)	1.21	0.65, 2.28	0.5
Rituximab maintenance yes (versus no)	1.05	0.60, 1.81	0.9
Treatment response to first line			
SD + PD	Reference		
PR	0.89	0.40, 1.98	0.8
CR/CRu	0.86	0.40, 1.84	0.7
POD after first line (continuous)	0.99	0.98, 1.00	0.2
POD12, yes (versus no)	1.53	0.78, 2.99	0.16
POD24, yes (versus no)	1.42	0.82, 2.47	0.2
POD36, (versus no)	0.99	0.52, 1.86	0.97
BM involvement before ibrutinib, yes (versus no)	0.65	0.32, 1.32	0.23
Lines before ibrutinib, 1 vs. 2 and more	0.49	0.27, 0.87	*0.035*
Treatment response to ibrutinib			
SD + PD	Reference		
PMR	0.56	0.30, 1.06	0.074
CMR	0.24	0.11, 0.52	< *0.001*
Univariate Cox regression analysis for overall survival calculated from diagnosis			
Female sex (versus male)	0.83	0.40, 1.71	0.6
Ki67 ≥ 30% (versus < 30%)	2.56	1.03, 6.37	*0.044*
Ann Arbor clinical stage			
I	Reference		
II	0.51	0.12, 2.07	0.3
III	0.88	0.22, 3.56	0.9
IV	0.97	0.34, 2.77	> 0.9
MIPI			
Low risk	Reference	—	
Intermediate risk	2.88	0.72, 11.6	0.14
High risk	3.71	1.07, 12.9	*0.039*
First line regimen			
CHOP/CHOP-like	Reference	—	
High-dose cytarabine regimen	0.96	0.51, 1.81	0.9
Non-anthracycline regimen	0.74	0.32, 1.70	0.5
ASCT, yes (versus no)	1.39	0.75, 2.55	0.3
Rituximab maintenance yes (versus no)	1	0.56, 1.77	> 0.9
Treatment response to first line			
SD + PD	Reference		
PR	0.28	0.12, 0.68	*0.005*
CR/CRu	0.15	0.06, 0.36	< *0.001*
POD after first line (continuous)	0.98	0.97, 0.99	*0.002*
POD12, yes (versus no)	2.99	1.64, 5.45	< *0.001*
POD24, yes (versus no)	2.87	1.57, 5.22	< *0.001*
POD36, yes (versus no)	2.08	1.07, 4.04	*0.03*
BM involvement before ibrutinib yes (versus no)	0.65	0.33, 1.28	0.2
Lines before ibrutinib	0.76	0.62, 0.94	*0.013*
Treatment response to ibrutinib			
SD + PD	Reference		
PMR	0.77	0.41, 1.46	0.4
CMR	0.39	0.18, 0.88	*0.022*
Multivariate Cox regression analysis for overall survival calculated from ibrutinib initiation			
Lines before ibrutinib	0.55	0.31, 0.95	*0.032*
Ki67 ≥ 30% (versus < 30%)	2.73	1.04, 7.15	*0.042*

MIPI, Mantle Cell Lymphoma International Prognostic Index; ASCT, autologous stem cell transplant; CR, complete remission; PR, partial remission; SD, stable disease; PD, progressive disease; CMR, complete metabolic remission; PMR, partial metabolic remission; POD, progression of disease; BM, bone marrow

According to multivariate logistic regression, high proliferation rate by Ki67 and response to ibrutinib proved to be independent predictors of OS (*p* = 0.032 and *p* = 0.042, respectively).

Until the database was locked, 56 (73%) patients had discontinued ibrutinib due to progression or relapse, four patients due to toxicity, and one patient at his own request with no specific reason. The reason for stopping ibrutinib in another one patient was unknown. Forty-five patients had died. Median PFS in patients, who progressed or relapsed on ibrutinib (after having achieved CMR or PMR) was 4.9 months (range: 1.2–54.5 months). The median OS of these patients from the date of ibrutinib failure was only 3.7 months (range: 0–5.8 months). Forty-two patients received palliative or best supportive therapy including bortezomib, lenalidomide, temsirolimus, or immunochemotherapy and succumbed to refractory MCL. From 11 patients who failed ibrutinib and were still alive at the time of the database lock, five received R-BAC500 (rituximab, bendamustine, and cytarabine), two were treated with venetoclax monotherapy, and the remaining four patients were treated with bortezomib (plus rituximab), single-agent lenalidomide, bendamustine (plus rituximab), and palliative immunochemotherapy. One patient was prepared for, and another one successfully underwent allogeneic stem cell transplantation.

## Discussion

Data on ibrutinib efficacy in unselected patients with R/R MCL outside prospective clinical trials are still limited. Several prospective clinical trials confirmed the efficacy of and long-term response to ibrutinib in R/R MCL and conduced to the wide use of ibrutinib also in heavily pre-treated unselected patients [23, 24, 26, 28, 29]. Results of both prospective and retrospective trials confirmed that the administration of ibrutinib is most beneficial in early lines of therapy [24, 26]. One-third of our patients received ibrutinib as a second-line therapy, which is consistent with current treatment recommendations [1]. Moreover, they achieved significantly longer OS compared to those with ibrutinib in later lines. Although McCulloch et al. analyzed patients after one previous line, an overall response rate (ORR) of less than 70% with the complete (CR/CRu) to partial remission (PR) ratio was similar to our results [26]. Comparable responses were also noted by Epperla et al. in a trial with almost similar characteristics of previous treatment [29]. Of note, also prospective trials by Dreyling et al. and by Wang et al. reported comparable ORRs [21, 33]. Kaplan–Meier survival estimates demonstrated slightly shorter OS and PFS in our cohort compared to other so far published studies. This discrepancy could be caused by more adverse demographic features such as more advanced age at the time of ibrutinib initiation or a larger proportion of patients with higher Ki67. In addition, selection bias compared to prospective trials might also be responsible for less favorable survival outcomes observed in our study [21, 22, 27–29].

Special attention should be focused on a study by Jain et al. The authors analyzed a relatively small cohort of 50 patients treated with a combination of ibrutinib and rituximab (IR). The outcomes were very promising in terms of efficacy, durable responses, and long-term survival. Compared to our results, patients treated with IR had more than 20% higher ORR and achieved almost 30% more CRs. Most importantly, the median PFS in IR-treated patients was four times longer compared to our data [34].

From the analyzed pre-treatment factors (i.e., before ibrutinib start), only proliferation rate by Ki67 correlated with outcome. Similar results were previously reported in other prospective and retrospective studies [26, 34].

An interesting finding, albeit not statistically significant, was identification of female gender as a predictor of better OS. Seventy-eight percent of patients in our retrospective analysis were males (4:1 ratio), almost double the proportion observed at diagnosis. In addition, the survival parameters of females were better than those of males, even though statistical significance was not reached. Previous studies reported on poorer MCL outcomes of males compared to females [35]. The observed differences could be caused by distinct effects of sex hormones on multiple extranodal tissues, sex-biased gene expression signatures, or differences in the genetic landscape between males and females at diagnosis [15, 36, 37]. This observation warrants further investigation.

According to recently published studies, the IR combination was highly effective in both newly diagnosed and R/R MCL patients [34, 38]. In our study, ibrutinib was not capable to effectively eliminate MCL cells from the BM of patients with detectable BM involvement before ibrutinib therapy. Several patients who achieved remission according to CT or PET/CT had even higher loads of MCL cells in BM. Of note, two patients experienced isolated progression of MCL in BM on ibrutinib therapy which led to therapy change. In one of these patients, addition of rituximab to ibrutinib resulted in rapid eradication of MCL cells in BM after as few as four weekly doses of rituximab. The proposed mode of action of the IR combination is plausibly based on a compartmental synergy, in which ibrutinib effectively eliminates nodal and extramedullary MCL cells, while rituximab effectively clears circulating MCL cells and MCL cells infiltrating the BM compartment.

Prognosis of the patients after ibrutinib failure was extremely poor regardless of the number of previous lines of therapy. Data on efficacy of post-ibrutinib regimens are limited, and no subsequent therapeutic standards have been established [39–44]. Treatment strategies after ibrutinib failures were highly diverse and depended on the center practice. None of the single agents like bortezomib, venetoclax, lenalidomide, or temsirolimus demonstrated satisfactory effect. From combined regimens, only R-BAC500, first investigated as a frontline regimen [39], conduced to satisfactory responses. All our patients who responded to R-BAC500 were alive more than nine months from ibrutinib failure and were still in remission. Our findings confirmed promising results of a study with R-BAC500 in R/R MCL by McCulloch et al. with 83% ORR (60% CR/CR unconfirmed) and a median OS of 12.5 months [40].
